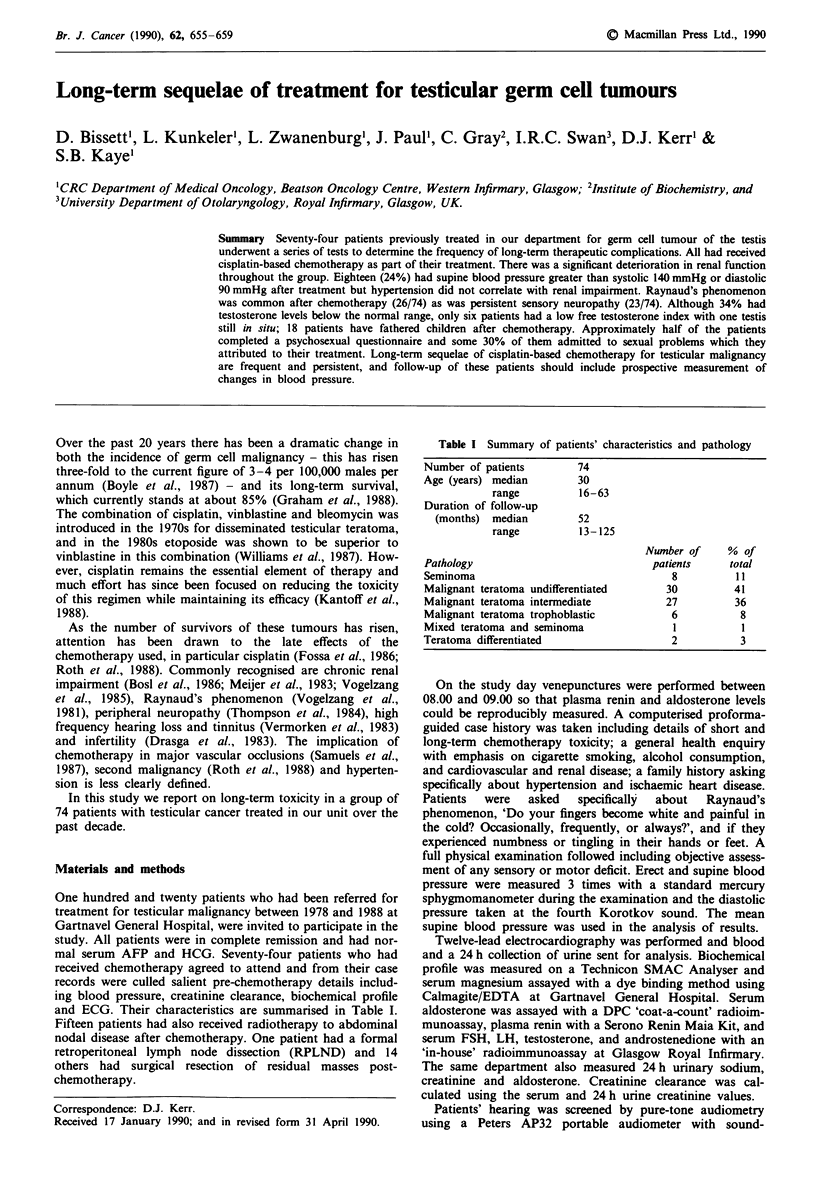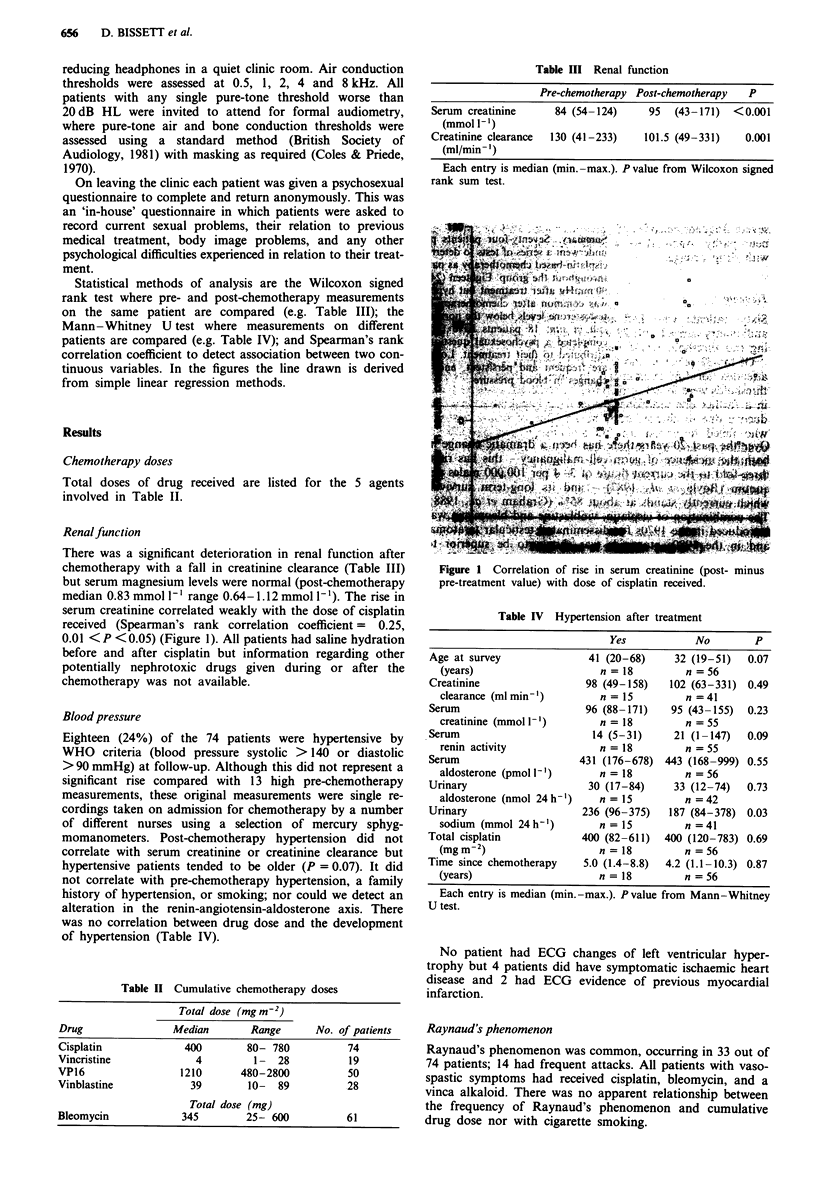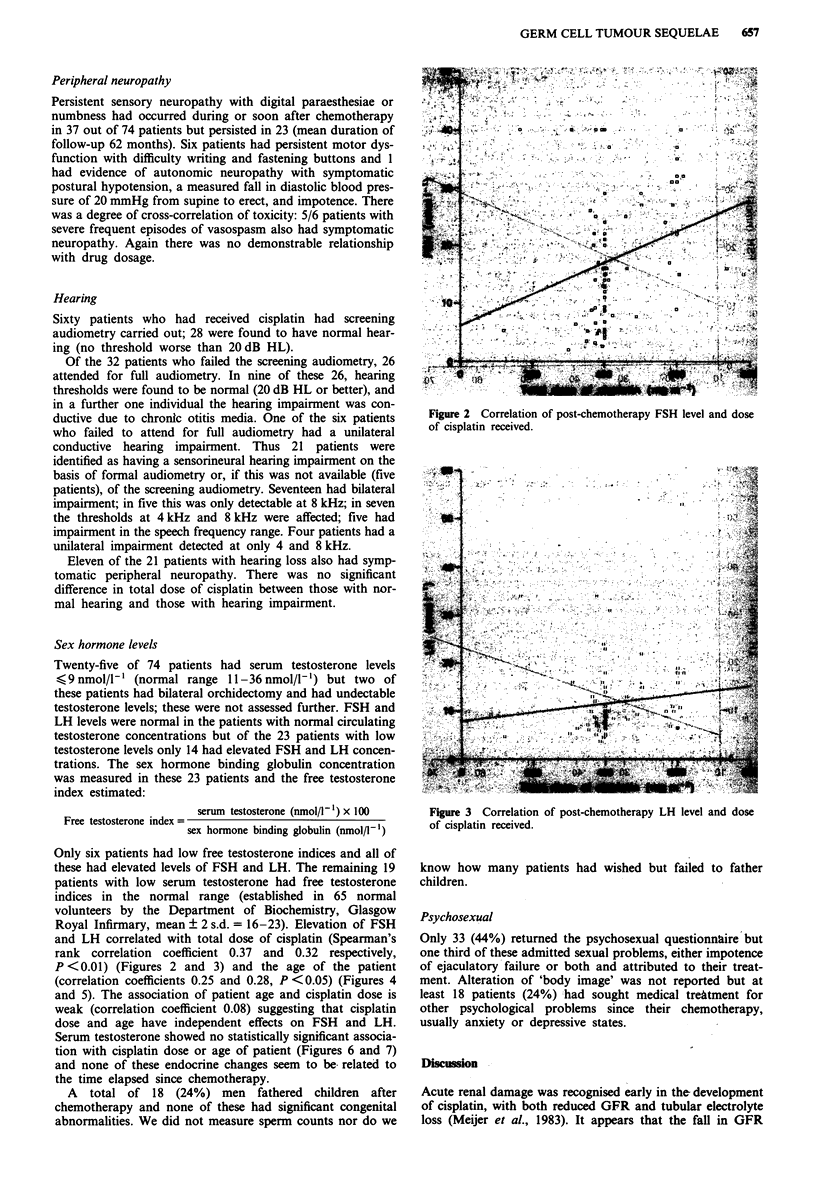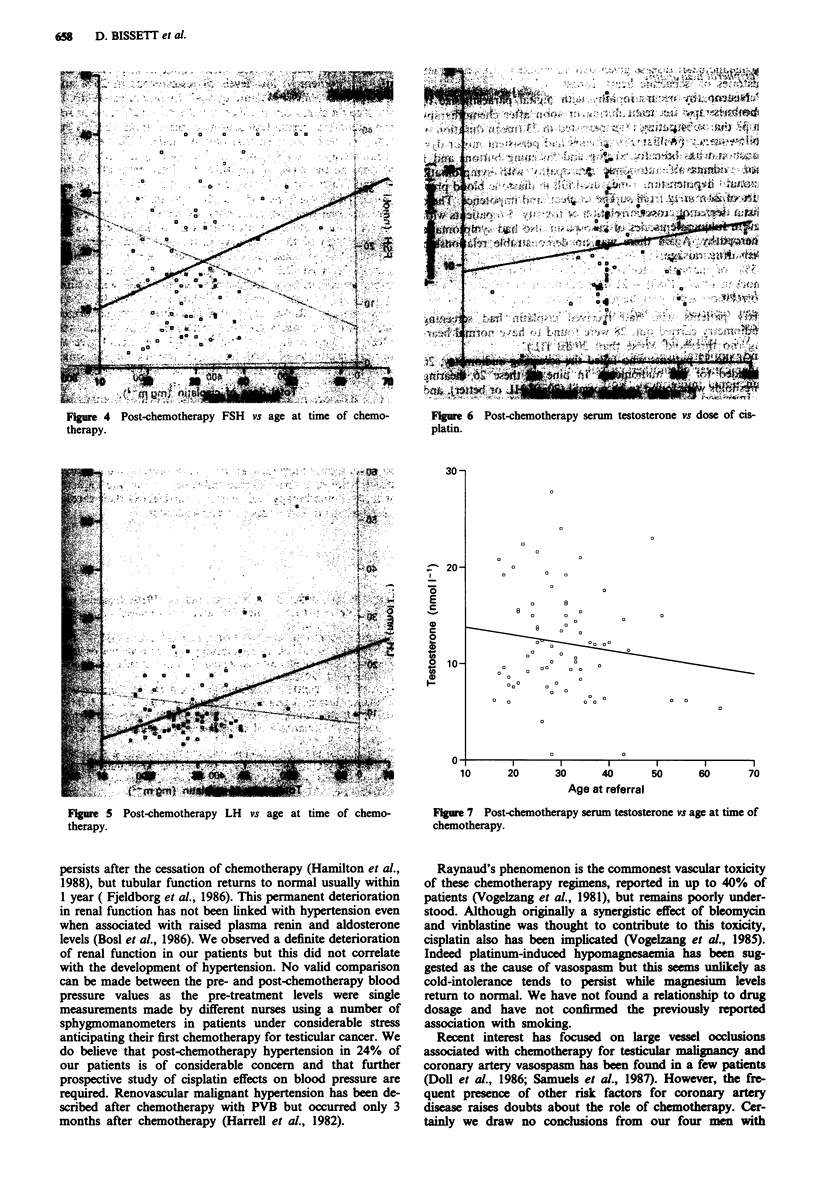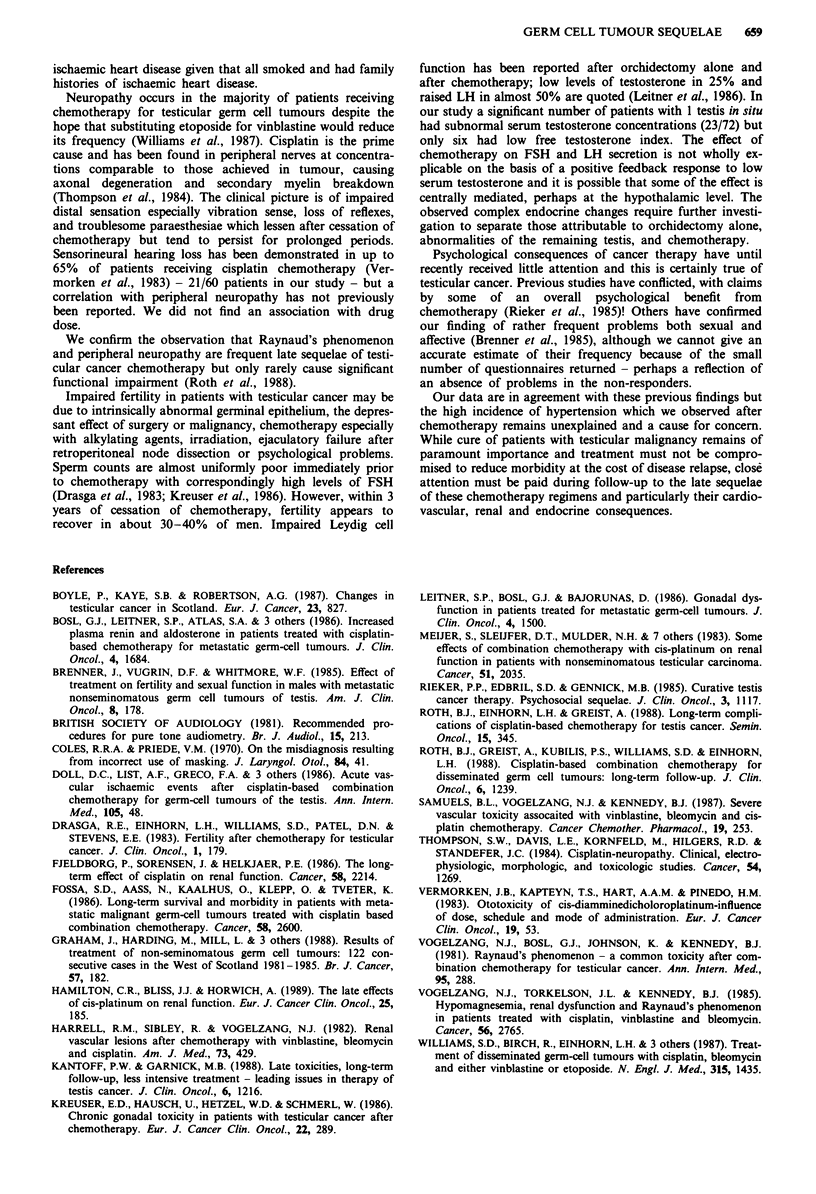# Long-term sequelae of treatment for testicular germ cell tumours.

**DOI:** 10.1038/bjc.1990.350

**Published:** 1990-10

**Authors:** D. Bissett, L. Kunkeler, L. Zwanenburg, J. Paul, C. Gray, I. R. Swan, D. J. Kerr, S. B. Kaye

**Affiliations:** CRC Department of Medical Oncology, Beatson Oncology Centre, Western Infirmary, Glasgow, UK.

## Abstract

Seventy-four patients previously treated in our department for germ cell tumour of the testis underwent a series of tests to determine the frequency of long-term therapeutic complications. All had received cisplatin-based chemotherapy as part of their treatment. There was a significant deterioration in renal function throughout the group. Eighteen (24%) had supine blood pressure greater than systolic 140 mmHg or diastolic 90 mmHg after treatment but hypertension did not correlate with renal impairment. Raynaud's phenomenon was common after chemotherapy (26/74) as was persistent sensory neuropathy (23/74). Although 34% had testosterone levels below the normal range, only six patients had a low free testosterone index with one testis still in situ; 18 patients have fathered children after chemotherapy. Approximately half of the patients completed a psychosexual questionnaire and some 30% of them admitted to sexual problems which they attributed to their treatment. Long-term sequelae of cisplatin-based chemotherapy for testicular malignancy are frequent and persistent, and follow-up of these patients should include prospective measurement of changes in blood pressure.


Br. J. Cancer (1990), 62, 655-659 © Macmillan Press Ltd., 1990

### Long-term sequelae of treatment for testicular germ cell tumours

D. Bissett[1], L. Kunkeler[1], L. Zwanenburg[1], J. Paul[1], C. Gray[2], I.R.C. Swan[3], D.J. Kerr[1] & S.B. Kaye[1]

[1]*CRC Department of Medical Oncology, Beatson Oncology Centre, Western Infirmary, Glasgow;* [2]*Institute of Biochemistry, and* [3]*University Department of Otolaryngology, Royal Infirmary, Glasgow, UK.*

**Summary** Seventy-four patients previously treated in our department for germ cell tumour of the testis underwent a series of tests to determine the frequency of long-term therapeutic complications. All had received cisplatin-based chemotherapy as part of their treatment. There was a significant deterioration in renal function throughout the group. Eighteen (24%) had supine blood pressure greater than systolic 140 mmHg or diastolic 90 mmHg after treatment but hypertension did not correlate with renal impairment. Raynaud's phenomenon was common after chemotherapy (26/74) as was persistent sensory neuropathy (23/74). Although 34% had testosterone levels below the normal range, only six patients had a low free testosterone index with one testis still *in situ*; 18 patients have fathered children after chemotherapy. Approximately half of the patients completed a psychosexual questionnaire and some 30% of them admitted to sexual problems which they attributed to their treatment. Long-term sequelae of cisplatin-based chemotherapy for testicular malignancy are frequent and persistent, and follow-up of these patients should include prospective measurement of changes in blood pressure.

Over the past 20 years there has been a dramatic change in both the incidence of germ cell malignancy – this has risen three-fold to the current figure of 3–4 per 100,000 males per annum (Boyle *et al.*, 1987) – and its long-term survival, which currently stands at about 85% (Graham *et al.*, 1988). The combination of cisplatin, vinblastine and bleomycin was introduced in the 1970s for disseminated testicular teratoma, and in the 1980s etoposide was shown to be superior to vinblastine in this combination (Williams *et al.*, 1987). However, cisplatin remains the essential element of therapy and much effort has since been focused on reducing the toxicity of this regimen while maintaining its efficacy (Kantoff *et al.*, 1988).

As the number of survivors of these tumours has risen, attention has been drawn to the late effects of the chemotherapy used, in particular cisplatin (Fossa *et al.*, 1986; Roth *et al.*, 1988). Commonly recognised are chronic renal impairment (Bosl *et al.*, 1986; Meijer *et al.*, 1983; Vogelzang *et al.*, 1985), Raynaud's phenomenon (Vogelzang *et al.*, 1981), peripheral neuropathy (Thompson *et al.*, 1984), high frequency hearing loss and tinnitus (Vermorken *et al.*, 1983) and infertility (Drasga *et al.*, 1983). The implication of chemotherapy in major vascular occlusions (Samuels *et al.*, 1987), second malignancy (Roth *et al.*, 1988) and hypertension is less clearly defined.

In this study we report on long-term toxicity in a group of 74 patients with testicular cancer treated in our unit over the past decade.

### Materials and methods

One hundred and twenty patients who had been referred for treatment for testicular malignancy between 1978 and 1988 at Gartnavel General Hospital, were invited to participate in the study. All patients were in complete remission and had normal serum AFP and HCG. Seventy-four patients who had received chemotherapy agreed to attend and from their case records were culled salient pre-chemotherapy details including blood pressure, creatinine clearance, biochemical profile and ECG. Their characteristics are summarised in Table I. Fifteen patients had also received radiotherapy to abdominal nodal disease after chemotherapy. One patient had a formal retroperitoneal lymph node dissection (RPLND) and 14 others had surgical resection of residual masses post-chemotherapy.

**Table I** Summary of patients' characteristics and pathology

| | | | |
|---|---|---|---|
| Number of patients | 74 | | |
| Age (years) median | 30 | | |
| range | 16–63 | | |
| Duration of follow-up (months) median | 52 | | |
| range | 13–125 | | |
| *Pathology* | | *Number of patients* | *% of total* |
| Seminoma | | 8 | 11 |
| Malignant teratoma undifferentiated | | 30 | 41 |
| Malignant teratoma intermediate | | 27 | 36 |
| Malignant teratoma trophoblastic | | 6 | 8 |
| Mixed teratoma and seminoma | | 1 | 1 |
| Teratoma differentiated | | 2 | 3 |

On the study day venepunctures were performed between 08.00 and 09.00 so that plasma renin and aldosterone levels could be reproducibly measured. A computerised proforma-guided case history was taken including details of short and long-term chemotherapy toxicity; a general health enquiry with emphasis on cigarette smoking, alcohol consumption, and cardiovascular and renal disease; a family history asking specifically about hypertension and ischaemic heart disease. Patients were asked specifically about Raynaud's phenomenon, 'Do your fingers become white and painful in the cold? Occasionally, frequently, or always?', and if they experienced numbness or tingling in their hands or feet. A full physical examination followed including objective assessment of any sensory or motor deficit. Erect and supine blood pressure were measured 3 times with a standard mercury sphygmomanometer during the examination and the diastolic pressure taken at the fourth Korotkov sound. The mean supine blood pressure was used in the analysis of results.

Twelve-lead electrocardiography was performed and blood and a 24 h collection of urine sent for analysis. Biochemical profile was measured on a Technicon SMAC Analyser and serum magnesium assayed with a dye binding method using Calmagite/EDTA at Gartnavel General Hospital. Serum aldosterone was assayed with a DPC 'coat-a-count' radioimmunoassay, plasma renin with a Serono Renin Maia Kit, and serum FSH, LH, testosterone, and androstenedione with an 'in-house' radioimmunoassay at Glasgow Royal Infirmary. The same department also measured 24 h urinary sodium, creatinine and aldosterone. Creatinine clearance was calculated using the serum and 24 h urine creatinine values.

Patients' hearing was screened by pure-tone audiometry using a Peters AP32 portable audiometer with sound-

Correspondence: D.J. Kerr.
Received 17 January 1990; and in revised form 31 April 1990.

reducing headphones in a quiet clinic room. Air conduction thresholds were assessed at 0.5, 1, 2, 4 and 8 kHz. All patients with any single pure-tone threshold worse than 20 dB HL were invited to attend for formal audiometry, where pure-tone air and bone conduction thresholds were assessed using a standard method (British Society of Audiology, 1981) with masking as required (Coles & Priede, 1970).

On leaving the clinic each patient was given a psychosexual questionnaire to complete and return anonymously. This was an 'in-house' questionnaire in which patients were asked to record current sexual problems, their relation to previous medical treatment, body image problems, and any other psychological difficulties experienced in relation to their treatment.

Statistical methods of analysis are the Wilcoxon signed rank test where pre- and post-chemotherapy measurements on the same patient are compared (e.g. Table III); the Mann–Whitney U test where measurements on different patients are compared (e.g. Table IV); and Spearman's rank correlation coefficient to detect association between two continuous variables. In the figures the line drawn is derived from simple linear regression methods.

### Results

### *Chemotherapy doses*

Total doses of drug received are listed for the 5 agents involved in Table II.

### *Renal function*

There was a significant deterioration in renal function after chemotherapy with a fall in creatinine clearance (Table III) but serum magnesium levels were normal (post-chemotherapy median 0.83 mmol $l^{-1}$ range 0.64–1.12 mmol $l^{-1}$). The rise in serum creatinine correlated weakly with the dose of cisplatin received (Spearman's rank correlation coefficient = 0.25, $0.01 < P < 0.05$) (Figure 1). All patients had saline hydration before and after cisplatin but information regarding other potentially nephrotoxic drugs given during or after the chemotherapy was not available.

### *Blood pressure*

Eighteen (24%) of the 74 patients were hypertensive by WHO criteria (blood pressure systolic >140 or diastolic >90 mmHg) at follow-up. Although this did not represent a significant rise compared with 13 high pre-chemotherapy measurements, these original measurements were single recordings taken on admission for chemotherapy by a number of different nurses using a selection of mercury sphygmomanometers. Post-chemotherapy hypertension did not correlate with serum creatinine or creatinine clearance but hypertensive patients tended to be older ($P = 0.07$). It did not correlate with pre-chemotherapy hypertension, a family history of hypertension, or smoking; nor could we detect an alteration in the renin-angiotensin-aldosterone axis. There was no correlation between drug dose and the development of hypertension (Table IV).

**Table II** Cumulative chemotherapy doses

| | Total dose ($mg\,m^{-2}$) | | |
|---|---|---|---|
| *Drug* | *Median* | *Range* | *No. of patients* |
| Cisplatin | 400 | 80– 780 | 74 |
| Vincristine | 4 | 1– 28 | 19 |
| VP16 | 1210 | 480–2800 | 50 |
| Vinblastine | 39 | 10– 89 | 28 |
| | *Total dose (mg)* | | |
| Bleomycin | 345 | 25– 600 | 61 |

**Table III** Renal function

| | *Pre-chemotherapy* | *Post-chemotherapy* | *P* |
|---|---|---|---|
| Serum creatinine (mmol $l^{-1}$) | 84 (54–124) | 95 (43–171) | <0.001 |
| Creatinine clearance (ml/$min^{-1}$) | 130 (41–233) | 101.5 (49–331) | 0.001 |

Each entry is median (min.–max.). *P* value from Wilcoxon signed rank sum test.

**Figure 1** Correlation of rise in serum creatinine (post- minus pre-treatment value) with dose of cisplatin received.

**Table IV** Hypertension after treatment

| | *Yes* | *No* | *P* |
|---|---|---|---|
| Age at survey (years) | 41 (20–68)<br>$n = 18$ | 32 (19–51)<br>$n = 56$ | 0.07 |
| Creatinine clearance (ml $min^{-1}$) | 98 (49–158)<br>$n = 15$ | 102 (63–331)<br>$n = 41$ | 0.49 |
| Serum creatinine (mmol $l^{-1}$) | 96 (88–171)<br>$n = 18$ | 95 (43–155)<br>$n = 55$ | 0.23 |
| Serum renin activity | 14 (5–31)<br>$n = 18$ | 21 (1–147)<br>$n = 55$ | 0.09 |
| Serum aldosterone (pmol $l^{-1}$) | 431 (176–678)<br>$n = 18$ | 443 (168–999)<br>$n = 56$ | 0.55 |
| Urinary aldosterone (nmol 24 $h^{-1}$) | 30 (17–84)<br>$n = 15$ | 33 (12–74)<br>$n = 42$ | 0.73 |
| Urinary sodium (mmol 24 $h^{-1}$) | 236 (96–375)<br>$n = 15$ | 187 (84–378)<br>$n = 41$ | 0.03 |
| Total cisplatin ($mg\,m^{-2}$) | 400 (82–611)<br>$n = 18$ | 400 (120–783)<br>$n = 56$ | 0.69 |
| Time since chemotherapy (years) | 5.0 (1.4–8.8)<br>$n = 18$ | 4.2 (1.1–10.3)<br>$n = 56$ | 0.87 |

Each entry is median (min.–max.). *P* value from Mann–Whitney U test.

No patient had ECG changes of left ventricular hypertrophy but 4 patients did have symptomatic ischaemic heart disease and 2 had ECG evidence of previous myocardial infarction.

### *Raynaud's phenomenon*

Raynaud's phenomenon was common, occurring in 33 out of 74 patients; 14 had frequent attacks. All patients with vasospastic symptoms had received cisplatin, bleomycin, and a vinca alkaloid. There was no apparent relationship between the frequency of Raynaud's phenomenon and cumulative drug dose nor with cigarette smoking.

*Peripheral neuropathy*

Persistent sensory neuropathy with digital paraesthesiae or numbness had occurred during or soon after chemotherapy in 37 out of 74 patients but persisted in 23 (mean duration of follow-up 62 months). Six patients had persistent motor dysfunction with difficulty writing and fastening buttons and 1 had evidence of autonomic neuropathy with symptomatic postural hypotension, a measured fall in diastolic blood pressure of 20 mmHg from supine to erect, and impotence. There was a degree of cross-correlation of toxicity: 5/6 patients with severe frequent episodes of vasospasm also had symptomatic neuropathy. Again there was no demonstrable relationship with drug dosage.

*Hearing*

Sixty patients who had received cisplatin had screening audiometry carried out; 28 were found to have normal hearing (no threshold worse than 20 dB HL).

Of the 32 patients who failed the screening audiometry, 26 attended for full audiometry. In nine of these 26, hearing thresholds were found to be normal (20 dB HL or better), and in a further one individual the hearing impairment was conductive due to chronic otitis media. One of the six patients who failed to attend for full audiometry had a unilateral conductive hearing impairment. Thus 21 patients were identified as having a sensorineural hearing impairment on the basis of formal audiometry or, if this was not available (five patients), of the screening audiometry. Seventeen had bilateral impairment; in five this was only detectable at 8 kHz; in seven the thresholds at 4 kHz and 8 kHz were affected; five had impairment in the speech frequency range. Four patients had a unilateral impairment detected at only 4 and 8 kHz.

Eleven of the 21 patients with hearing loss also had symptomatic peripheral neuropathy. There was no significant difference in total dose of cisplatin between those with normal hearing and those with hearing impairment.

*Sex hormone levels*

Twenty-five of 74 patients had serum testosterone levels $\leqslant 9\ nmol/l^{-1}$ (normal range $11-36\ nmol/l^{-1}$) but two of these patients had bilateral orchidectomy and had undectable testosterone levels; these were not assessed further. FSH and LH levels were normal in the patients with normal circulating testosterone concentrations but of the 23 patients with low testosterone levels only 14 had elevated FSH and LH concentrations. The sex hormone binding globulin concentration was measured in these 23 patients and the free testosterone index estimated:

$$\text{Free testosterone index} = \frac{\text{serum testosterone (nmol/l}^{-1}) \times 100}{\text{sex hormone binding globulin (nmol/l}^{-1})}$$

Only six patients had low free testosterone indices and all of these had elevated levels of FSH and LH. The remaining 19 patients with low serum testosterone had free testosterone indices in the normal range (established in 65 normal volunteers by the Department of Biochemistry, Glasgow Royal Infirmary, mean ± 2 s.d. = 16–23). Elevation of FSH and LH correlated with total dose of cisplatin (Spearman's rank correlation coefficient 0.37 and 0.32 respectively, $P < 0.01$) (Figures 2 and 3) and the age of the patient (correlation coefficients 0.25 and 0.28, $P < 0.05$) (Figures 4 and 5). The association of patient age and cisplatin dose is weak (correlation coefficient 0.08) suggesting that cisplatin dose and age have independent effects on FSH and LH. Serum testosterone showed no statistically significant association with cisplatin dose or age of patient (Figures 6 and 7) and none of these endocrine changes seem to be related to the time elapsed since chemotherapy.

A total of 18 (24%) men fathered children after chemotherapy and none of these had significant congenital abnormalities. We did not measure sperm counts nor do we know how many patients had wished but failed to father children.

**Figure 2** Correlation of post-chemotherapy FSH level and dose of cisplatin received.

**Figure 3** Correlation of post-chemotherapy LH level and dose of cisplatin received.

*Psychosexual*

Only 33 (44%) returned the psychosexual questionnaire but one third of these admitted sexual problems, either impotence of ejaculatory failure or both and attributed to their treatment. Alteration of 'body image' was not reported but at least 18 patients (24%) had sought medical treatment for other psychological problems since their chemotherapy, usually anxiety or depressive states.

### Discussion

Acute renal damage was recognised early in the development of cisplatin, with both reduced GFR and tubular electrolyte loss (Meijer *et al.*, 1983). It appears that the fall in GFR

**Figure 4** Post-chemotherapy FSH vs age at time of chemotherapy.

**Figure 5** Post-chemotherapy LH vs age at time of chemotherapy.

**Figure 6** Post-chemotherapy serum testosterone vs dose of cisplatin.

**Figure 7** Post-chemotherapy serum testosterone vs age at time of chemotherapy.

persists after the cessation of chemotherapy (Hamilton et al., 1988), but tubular function returns to normal usually within 1 year ( Fjeldborg et al., 1986). This permanent deterioration in renal function has not been linked with hypertension even when associated with raised plasma renin and aldosterone levels (Bosl et al., 1986). We observed a definite deterioration of renal function in our patients but this did not correlate with the development of hypertension. No valid comparison can be made between the pre- and post-chemotherapy blood pressure values as the pre-treatment levels were single measurements made by different nurses using a number of sphygmomanometers in patients under considerable stress anticipating their first chemotherapy for testicular cancer. We do believe that post-chemotherapy hypertension in 24% of our patients is of considerable concern and that further prospective study of cisplatin effects on blood pressure are required. Renovascular malignant hypertension has been described after chemotherapy with PVB but occurred only 3 months after chemotherapy (Harrell et al., 1982).

Raynaud's phenomenon is the commonest vascular toxicity of these chemotherapy regimens, reported in up to 40% of patients (Vogelzang et al., 1981), but remains poorly understood. Although originally a synergistic effect of bleomycin and vinblastine was thought to contribute to this toxicity, cisplatin also has been implicated (Vogelzang et al., 1985). Indeed platinum-induced hypomagnesaemia has been suggested as the cause of vasospasm but this seems unlikely as cold-intolerance tends to persist while magnesium levels return to normal. We have not found a relationship to drug dosage and have not confirmed the previously reported association with smoking.

Recent interest has focused on large vessel occlusions associated with chemotherapy for testicular malignancy and coronary artery vasospasm has been found in a few patients (Doll et al., 1986; Samuels et al., 1987). However, the frequent presence of other risk factors for coronary artery disease raises doubts about the role of chemotherapy. Certainly we draw no conclusions from our four men with ischaemic heart disease given that all smoked and had family histories of ischaemic heart disease.

Neuropathy occurs in the majority of patients receiving chemotherapy for testicular germ cell tumours despite the hope that substituting etoposide for vinblastine would reduce its frequency (Williams *et al*., 1987). Cisplatin is the prime cause and has been found in peripheral nerves at concentrations comparable to those achieved in tumour, causing axonal degeneration and secondary myelin breakdown (Thompson *et al*., 1984). The clinical picture is of impaired distal sensation especially vibration sense, loss of reflexes, and troublesome paraesthesiae which lessen after cessation of chemotherapy but tend to persist for prolonged periods. Sensorineural hearing loss has been demonstrated in up to 65% of patients receiving cisplatin chemotherapy (Vermorken *et al*., 1983) – 21/60 patients in our study – but a correlation with peripheral neuropathy has not previously been reported. We did not find an association with drug dose.

We confirm the observation that Raynaud's phenomenon and peripheral neuropathy are frequent late sequelae of testicular cancer chemotherapy but only rarely cause significant functional impairment (Roth *et al*., 1988).

Impaired fertility in patients with testicular cancer may be due to intrinsically abnormal germinal epithelium, the depressant effect of surgery or malignancy, chemotherapy especially with alkylating agents, irradiation, ejaculatory failure after retroperitoneal node dissection or psychological problems. Sperm counts are almost uniformly poor immediately prior to chemotherapy with correspondingly high levels of FSH (Drasga *et al*., 1983; Kreuser *et al*., 1986). However, within 3 years of cessation of chemotherapy, fertility appears to recover in about 30–40% of men. Impaired Leydig cell function has been reported after orchidectomy alone and after chemotherapy; low levels of testosterone in 25% and raised LH in almost 50% are quoted (Leitner *et al*., 1986). In our study a significant number of patients with 1 testis *in situ* had subnormal serum testosterone concentrations (23/72) but only six had low free testosterone index. The effect of chemotherapy on FSH and LH secretion is not wholly explicable on the basis of a positive feedback response to low serum testosterone and it is possible that some of the effect is centrally mediated, perhaps at the hypothalamic level. The observed complex endocrine changes require further investigation to separate those attributable to orchidectomy alone, abnormalities of the remaining testis, and chemotherapy.

Psychological consequences of cancer therapy have until recently received little attention and this is certainly true of testicular cancer. Previous studies have conflicted, with claims by some of an overall psychological benefit from chemotherapy (Rieker *et al*., 1985)! Others have confirmed our finding of rather frequent problems both sexual and affective (Brenner *et al*., 1985), although we cannot give an accurate estimate of their frequency because of the small number of questionnaires returned – perhaps a reflection of an absence of problems in the non-responders.

Our data are in agreement with these previous findings but the high incidence of hypertension which we observed after chemotherapy remains unexplained and a cause for concern. While cure of patients with testicular malignancy remains of paramount importance and treatment must not be compromised to reduce morbidity at the cost of disease relapse, close attention must be paid during follow-up to the late sequelae of these chemotherapy regimens and particularly their cardiovascular, renal and endocrine consequences.

### References

BOYLE, P., KAYE, S.B. & ROBERTSON, A.G. (1987). Changes in testicular cancer in Scotland. *Eur. J. Cancer*, **23**, 827.

BOSL, G.J., LEITNER, S.P., ATLAS, S.A. & 3 others (1986). Increased plasma renin and aldosterone in patients treated with cisplatin-based chemotherapy for metastatic germ-cell tumours. *J. Clin. Oncol*., **4**, 1684.

BRENNER, J., VUGRIN, D.F. & WHITMORE, W.F. (1985). Effect of treatment on fertility and sexual function in males with metastatic nonseminomatous germ cell tumours of testis. *Am. J. Clin. Oncol*., **8**, 178.

BRITISH SOCIETY OF AUDIOLOGY (1981). Recommended procedures for pure tone audiometry. *Br. J. Audiol*., **15**, 213.

COLES, R.R.A. & PRIEDE, V.M. (1970). On the misdiagnosis resulting from incorrect use of masking. *J. Laryngol. Otol*., **84**, 41.

DOLL, D.C., LIST, A.F., GRECO, F.A. & 3 others (1986). Acute vascular ischaemic events after cisplatin-based combination chemotherapy for germ-cell tumours of the testis. *Ann. Intern. Med*., **105**, 48.

DRASGA, R.E., EINHORN, L.H., WILLIAMS, S.D., PATEL, D.N. & STEVENS, E.E. (1983). Fertility after chemotherapy for testicular cancer. *J. Clin. Oncol*., **1**, 179.

FJELDBORG, P., SORENSEN, J. & HELKJAER, P.E. (1986). The long-term effect of cisplatin on renal function. *Cancer*, **58**, 2214.

FOSSA, S.D., AASS, N., KAALHUS, O., KLEPP, O. & TVETER, K. (1986). Long-term survival and morbidity in patients with metastatic malignant germ-cell tumours treated with cisplatin based combination chemotherapy. *Cancer*, **58**, 2600.

GRAHAM, J., HARDING, M., MILL, L. & 3 others (1988). Results of treatment of non-seminomatous germ cell tumours: 122 consecutive cases in the West of Scotland 1981–1985. *Br. J. Cancer*, **57**, 182.

HAMILTON, C.R., BLISS, J.J. & HORWICH, A. (1989). The late effects of cis-platinum on renal function. *Eur. J. Cancer Clin. Oncol*., **25**, 185.

HARRELL, R.M., SIBLEY, R. & VOGELZANG, N.J. (1982). Renal vascular lesions after chemotherapy with vinblastine, bleomycin and cisplatin. *Am. J. Med*., **73**, 429.

KANTOFF, P.W. & GARNICK, M.B. (1988). Late toxicities, long-term follow-up, less intensive treatment – leading issues in therapy of testis cancer. *J. Clin. Oncol*., **6**, 1216.

KREUSER, E.D., HAUSCH, U., HETZEL, W.D. & SCHMERL, W. (1986). Chronic gonadal toxicity in patients with testicular cancer after chemotherapy. *Eur. J. Cancer Clin. Oncol*., **22**, 289.

LEITNER, S.P., BOSL, G.J. & BAJORUNAS, D. (1986). Gonadal dysfunction in patients treated for metastatic germ-cell tumours. *J. Clin. Oncol*., **4**, 1500.

MEIJER, S., SLEIJFER, D.T., MULDER, N.H. & 7 others (1983). Some effects of combination chemotherapy with cis-platinum on renal function in patients with nonseminomatous testicular carcinoma. *Cancer*, **51**, 2035.

RIEKER, P.P., EDBRIL, S.D. & GENNICK, M.B. (1985). Curative testis cancer therapy. Psychosocial sequelae. *J. Clin. Oncol*., **3**, 1117.

ROTH, B.J., EINHORN, L.H. & GREIST, A. (1988). Long-term complications of cisplatin-based chemotherapy for testis cancer. *Semin. Oncol*., **15**, 345.

ROTH, B.J., GREIST, A., KUBILIS, P.S., WILLIAMS, S.D. & EINHORN, L.H. (1988). Cisplatin-based combination chemotherapy for disseminated germ cell tumours: long-term follow-up. *J. Clin. Oncol*., **6**, 1239.

SAMUELS, B.L., VOGELZANG, N.J. & KENNEDY, B.J. (1987). Severe vascular toxicity assocaited with vinblastine, bleomycin and cisplatin chemotherapy. *Cancer Chemother. Pharmacol*., **19**, 253.

THOMPSON, S.W., DAVIS, L.E., KORNFELD, M., HILGERS, R.D. & STANDEFER, J.C. (1984). Cisplatin-neuropathy. Clinical, electrophysiologic, morphologic, and toxicologic studies. *Cancer*, **54**, 1269.

VERMORKEN, J.B., KAPTEYN, T.S., HART, A.A.M. & PINEDO, H.M. (1983). Ototoxicity of cis-diamminedicholoroplatinum-influence of dose, schedule and mode of administration. *Eur. J. Cancer Clin. Oncol*., **19**, 53.

VOGELZANG, N.J., BOSL, G.J., JOHNSON, K. & KENNEDY, B.J. (1981). Raynaud's phenomenon – a common toxicity after combination chemotherapy for testicular cancer. *Ann. Intern. Med*., **95**, 288.

VOGELZANG, N.J., TORKELSON, J.L. & KENNEDY, B.J. (1985). Hypomagnesemia, renal dysfunction and Raynaud's phenomenon in patients treated with cisplatin, vinblastine and bleomycin. *Cancer*, **56**, 2765.

WILLIAMS, S.D., BIRCH, R., EINHORN, L.H. & 3 others (1987). Treatment of disseminated germ-cell tumours with cisplatin, bleomycin and either vinblastine or etoposide. *N. Engl. J. Med*., **315**, 1435.